# Study on prestress loss and safety performance of retard-bonded prestressed cast-in-place piles

**DOI:** 10.1371/journal.pone.0352608

**Published:** 2026-08-14

**Authors:** Wenwei Zhu, Xuelin Yang, Haoyi Zhou, Jingli Yang, Xinlin Yao

**Affiliations:** 1 Zhejiang Province Institute of Architectural Design and Research, Hangzhou, Zhejiang, China; 2 School of Civil Engineering, Xi’an University of Architecture & Technology, Xi’an, Shanxi, China; 3 Zhejiang Geologic and Mineral Resources Construction Co., Ltd., Hangzhou, Zhejiang, China; China Construction Fourth Engineering Division Corp. Ltd, CHINA

## Abstract

The retard-bonded prestressed cast-in-place pile (RBPCP) represents a novel anti-floating structural technology with prominent advantages, including low cost and improved crack resistance. The structure and design methodology of the RBPCP and a new arrangement form for the retard-bonded prestressed reinforcing bar (RBPR) were presented. The test equipment and two-stage loading method were developed to facilitate static uplifting load tests of RBPCPs. Test study and numerical analysis on the prestress loss were conducted. The results indicate that the prestress loss in the pile increases with pile depth. Comparative analysis between required prestress and reserve prestress indicates that such loss exerts a negligible influence on the lower pile part. Moreover, the entire pile can maintain a compressive stress state as long as the tension load reaches the designed uplift bearing capacity. Therefore, the RBPCP demonstrates outstanding safety performance and is suitable for widespread application in engineering.

## 1. Introduction

The development of urban underground spaces in China seeks to fulfill the public’s desire for an improved quality of life and has emerged as a significant driver of urban growth. In recent years, large-scale underground building complexes have been developed in coastal cities, and the issue of water buoyancy has consistently challenged the engineering community, remaining without an ideal solution. The anti-floating design has emerged as a limiting factor in the advancement of underground engineering [1–5]. Uplift cast-in-place piles serve as a prevalent method for preventing flotation. Nevertheless, the inadequate tensile strength of concrete makes the pile susceptible to cracking. Groundwater containing corrosive trace elements can infiltrate these cracks, causing corrosion of internal steel bars and the expansion of rusted concrete. This significantly jeopardizes the durability of components and may ultimately lead to anti-floating failure. Conventional uplift cast-in-place piles typically necessitate a significant quantity of reinforcing bars to manage pile cracking. In regions characterized by high groundwater levels, corrosive groundwater, or significant projects designed for a service life of 100 years, the durability of uplift piles cannot be assured, even when measures like increasing the reinforcement ratio are implemented, despite the economic implications.

Prestressing technology fundamentally addresses the inherent deficiency of concrete in tensile resistance. Based on different construction techniques, prestressed reinforcing bar can be classified into bonded [6–10], partially bonded [11–15], unbonded [16–20], and retard-bonded types [21–25]. Prestressed cast-in-place piles adopting the first three prestressed reinforcing bar types all have distinct drawbacks: bonded prestressed cast-in-place piles feature complicated construction procedures and high overall costs; partially bonded and unbonded prestressed cast-in-place piles rely heavily on end anchorage systems, and the anchorage zones require rigorous anti-corrosion protection, which introduces potential hidden durability risks. To overcome the above limitations of existing prestressed uplift pile systems, this study innovatively introduces retard-bonded prestressing technology into cast-in-place piles and develops a novel retard-bonded prestressed cast-in-place pile (RBPCP). Compared with all conventional prestressed cast-in-place piles, the proposed RBPCP retains the core merit of active crack control of prestressed concrete while completely eliminating the strong dependence on end anchorage systems found in unbonded and partially bonded prestressed piles. Meanwhile, high-strength retard-bonded prestressed reinforcing bars (RBPRs) are adopted to replace ordinary reinforcing bars. The effective tensile strength of reinforcing bars is increased from 150 ~ 200 MPa (with a material utilization ratio of 42% ~ 56%) in traditional cast-in-place piles to 1100 ~ 1340 MPa. This not only greatly improves the crack control performance of the pile shaft but also significantly reduces the construction cost of a single pile, resolving the engineering challenge of excessive costs for bonded prestressed cast-in-place piles.

The RBPCP, being a newly developed technology in recent years [26], has relatively limited results from studies and engineering applications. Unlike prestressed components that are tensioned on the ground [27–31], the RBPCP is influenced by the surrounding soil layers during the tensioning process, leading to ambiguity regarding the distribution of the effective prestress established along the pile. There is currently a lack of relevant research, indicating the need to investigate the loss of prestress in the RBPCP.

This study presents the configuration of the RBPCP and proposes the arrangement of retard-bonded prestressed reinforcing bar (RBPR), together with the corresponding design method. The test equipment and two-stage loading method tailored for the static uplift test of RBPCPs are comprehensively elaborated. Based on a practical engineering project, experimental investigations are performed to explore the prestress loss characteristics of RBPCPs, and the prestress distribution law is accordingly determined. Numerical calculations are further conducted to analyze the prestress loss and required prestress of RBPCPs, and the underlying mechanical principles are summarized, providing a reliable reference for practical engineering applications.

## 2. Composition, RBPR arrangement, and design method of RBPCP

### 2.1. Composition

**Fig 1:**
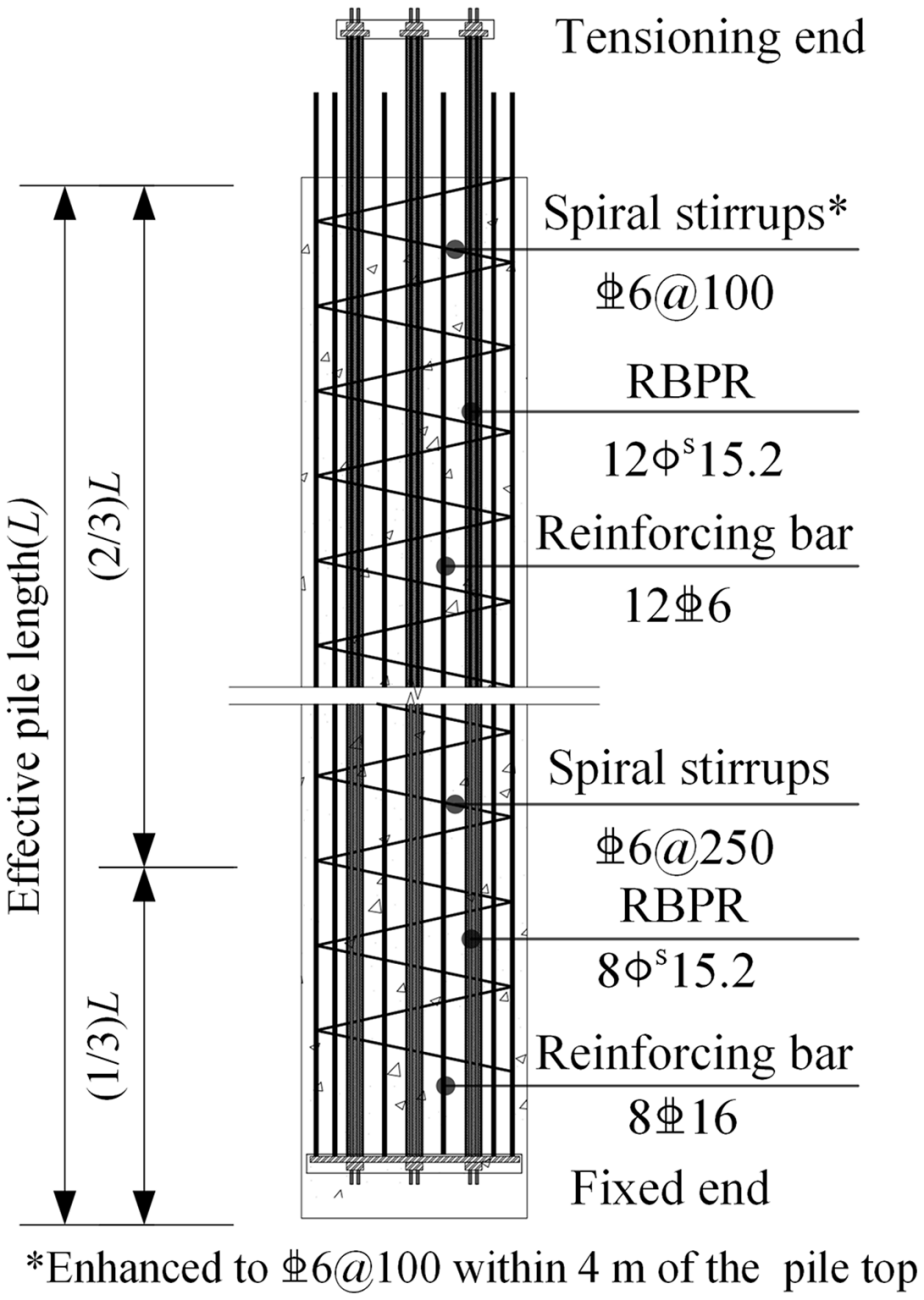
The diagram of the retard-bonded prestressed cast-in-place pile.

**Fig 2:**
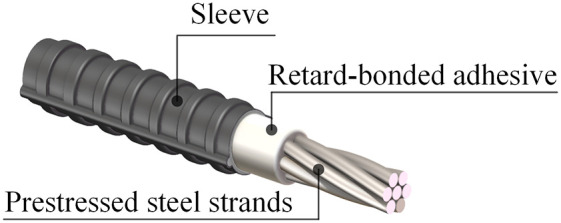
The retard-bonded prestressed reinforcing bar diagram.

Fig 1 illustrates that an RBPCP consists of multiple RBPRs, a standard reinforcing cage, and a concrete pile body. RBPRs consist of prestressed steel strands that are coated with a retard-bonded adhesive [32–36] and encased in a sleeve, as shown in Fig 2. The upper steel strands of each RBPR extend from the sleeve and connect to the anchor at the tensioning end, whereas the lower steel strands are attached to the reinforcing cage via a ring-shaped steel plate, functioning as the fixed end.

The tensioning process of the RBPCP is to be conducted following the excavation of the foundation pit. The construction duration for pile foundation engineering and earth excavation can be lengthy, often spanning several months. To accommodate the varying tensioning period requirements of different projects, it is advisable to choose retard-bonded adhesives that have distinct curing times.

Following the prestress tensioning and before the curing of the retard-bonded adhesive, the prestress of the steel strand is conveyed to the concrete of the pile body via the anchor and the fixed ring-shaped steel plate. Upon the curing of the retard-bonded adhesive, the steel strand achieves a tight connection with the concrete of the pile body, resulting in a cohesive force-bearing system akin to that of bonded prestressed cast-in-place piles. This addresses the limitations of bonded prestressed cast-in-place piles, which struggle with achieving effective bonding through pressure grouting, as well as unbonded prestressed cast-in-place piles, which experience prestress failure upon anchor failure.

### 2.2. RBPR arrangement

The arrangement of RBPR is associated with the production method of standard reinforcing cages:

(1) When the reinforcing cage is constructed as a single unit, the RBPR can be directly attached to the inner surface of the reinforcing cage. This method requires high standards for the construction site, working surface, and hoisting equipment, making it appropriate for projects in open site conditions.
(2) When the reinforcing cage is assembled by hoisting and connecting sections, it is essential to bind and fabricate the cage on-site at the opening of the pile hole. This process ensures the structural configuration (with closed stirrups on the exterior and steel strands on the interior), thereby securing the bearing capacity of the RBPCP. This method involves a complex construction process, an extended construction duration, and elevated labor expenses.

**Fig 3:**
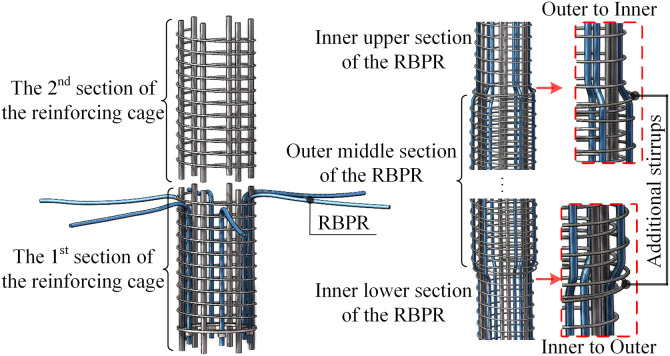
The RBPR arrangement.

Currently, splicing technology is extensively utilized in reinforcing cages. This paper proposes an arrangement form of RBPR, featuring an external configuration in the middle section and internal placements at both ends, as illustrated in Fig 3:

(1) Before hoisting the initial section of the reinforcing cage, the RBPRs are secured to the inner side of the bottom of the reinforcing cage. The extent of the first section of the steel reinforcing cage corresponds to the “inner lower section” of the RBPRs.
(2) When hoisting the second section of the reinforcing cage, the RBPRs are transferred from the inner side to the outer side of the reinforcing cage at the top of the first section. When hoisting the second and subsequent sections of the reinforcing cage, all RBPRs are positioned on the outer side of the cage. The stability of this section can only be maintained by placing additional stirrups on the outer side of the RBPRs. This section is referred to as the “outer middle section” of the RBPRs.
(3) When hoisting the final section of the reinforcing cage, the RBPRs are transferred from the outer side to the inner side of the reinforcing cage at a depth of 5m below the top of the reinforcing cage. The area extending 5m from the apex of the reinforcing cage is designated as the “inner upper section” of the RBPRs.

The new arrangement form, featuring an external section in the middle and internal sections at both ends, streamlines the construction process while maintaining the bearing capacity of RBPCP, resulting in significant savings in labor and time costs [37].

### 2.3. Design method

In the design of the RBPCP, it is essential to perform the uplift bearing capacity check and the pile body crack check in accordance with Equations (1) and (2), respectively, using the first-level crack control grade as a reference:


$$\begin{array}{c} N\leq f_{\text{y}}A_{\text{s}}+f_{\text{py}}A_{\text{p}} \end{array}$$ (1)

$$\begin{array}{c} \sigma_{\text{t}}=\frac{N_{\text{k}}}{A_{\text{0}}}-\frac{N_{p}-N_{l}}{A_{\text{n}}}\leq 0 \end{array}$$ (2)

$$\begin{array}{c} A_{\text{0}}=A_{\text{c}}+\alpha_{\text{E}}A_{\text{s}}+\alpha_{\text{Ep}}A_{\text{p}} \end{array}$$ (3)

$$\begin{array}{c} A_{\text{n}}=A_{\text{c}}+\alpha_{\text{E}}A_{\text{s}} \end{array}$$ (4)

Where *N* represents the design value of the axial tension at the pile top (N); *f*_y_ denotes the design value of the tensile strength of ordinary reinforcing bars (N/mm^2^); *f*_py_ indicates the design value of the tensile strength of steel strands (N/mm^2^); *A*_s_ refers to the cross-sectional area of ordinary reinforcing bars (mm^2^); *A*_p_ signifies the cross-sectional area of steel strands (mm^2^); *σ*_t_ is the tensile stress of pile body concrete (N/mm^2^); *N*_k_ is the axial tension value at the pile top under the standard combination (N); *A*_0_ represents the equivalent cross-sectional area of concrete, ordinary reinforcing bars, and steel strands (mm^2^); *A*_n_ denotes the equivalent cross-sectional area of concrete and ordinary reinforcing bars (mm^2^); *A*_c_ is the cross-sectional area of concrete (mm^2^); and *α*_E_ is the ratio of the elastic modulus of reinforcing bar to that of concrete; *α*_Ep_ represents the ratio of the elastic modulus of steel strand to that of concrete. *N*_p_ represents the design value of the tensile force (N), while *N*_*l*_ indicates the loss of prestress value for post-tensioned components (N) [38].

## 3. Equipment and loading method for the static uplifting load test of RBPCP

The pulling object of the static uplifting load test for traditional cast-in-place piles consists of ordinary reinforcing bars, whereas for RBPCPs, it includes RBPRs and ordinary reinforcing bars. In the course of the static uplifting load test, the full length of the RBPR is permitted to extend freely, whereas only the exposed end of the ordinary reinforcing bars is allowed to extend. When both are loaded at the same time, the standard reinforcing bars will inevitably fail beforehand, failing the test. This paper presents the development of specialized test equipment and a two-stage loading method designed for the static uplifting load test of the RBPCP.

**Fig 4:**
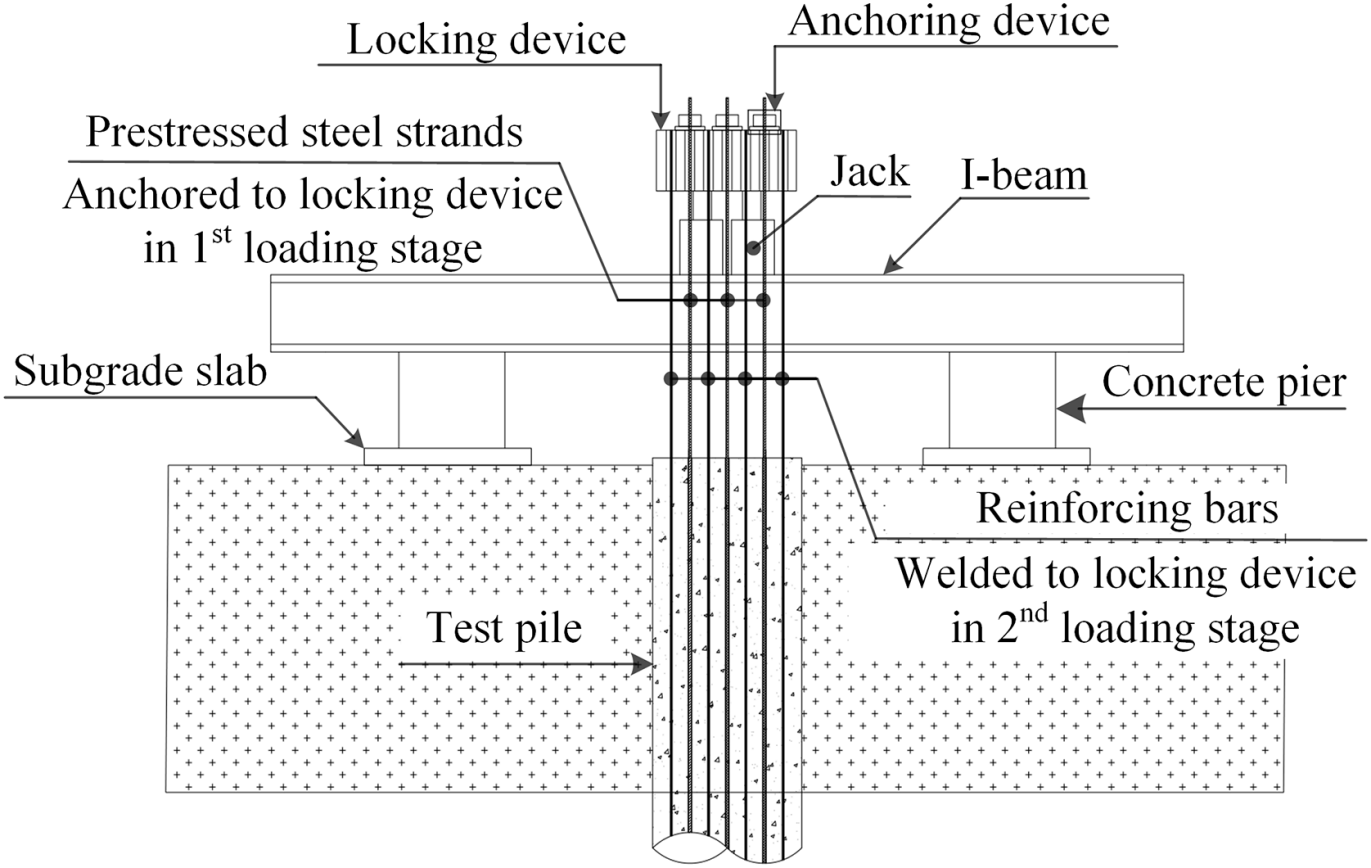
The apparatus employed for conducting the static uplifting load test of RBPCP.

The test equipment for the RBPCP, as illustrated in Fig 4, comprises subgrade slabs, concrete piers, I-beams, jacks, locking devices, and anchoring devices.

The detailed procedure for the two-stage loading method is outlined below:

(1) Initial loading stage: Secure the steel strands to the I-beam, maintain the ordinary reinforcing bars in an unrestrained condition, and exclusively apply load to the steel strands using the jack during this phase.
(2) Second loading stage: Upon reaching the predetermined elongation of the steel strands, proceed to weld the standard reinforcing bars to the I-beam, simultaneously loading both the standard reinforcing bars and the steel strands during this phase.

The procedure for establishing the preset elongation of the steel strands (Δ*L*) is as follows:


$$\begin{array}{c} \Delta L=\frac{Q_{\text{1}}\times L_{\text{p}}}{A_{\text{p}}E_{\text{p}}} \end{array}$$ (5)

$$\begin{array}{c} Q_{\text{1}}=Q-Q_{\text{2}} \end{array}$$ (6)

$$\begin{array}{c} Q_{\text{2}}=f_{\text{y}}A_{\text{s}}\left(\text{1 +}\frac{E_{\text{p}}A_{\text{p}}}{E_{\text{s}}A_{\text{s}}}\cdot \frac{L_{\text{s}}}{L_{\text{p}}}\right) \end{array}$$ (7)

Where *Q*_1_ represents the load value applied during the first stage (N); *L*_p_ denotes the total length of the prestressed steel strands (mm); *E*_p_ indicates the elastic modulus of the prestressed steel strands (MPa); *Q* refers to the maximum loading value necessary for the static uplifting load test (N); *Q*_2_ signifies the load value in the second stage (N); *L*_s_ is the exposed length of the ordinary reinforcing bar at the pile head (m).

## 4. Experimental study on prestress loss of RBPCP

### 4.1. Experimental purpose

In typical prestressed concrete elements tensioned at ground level, the factors contributing to prestress loss encompass anchor deformation, friction, relaxation of prestressed reinforcing bars, as well as shrinkage, creep, and elastic compression of the concrete, among others. The prestress loss resulting from all of the above is a fundamental part of the tensioning process, and the ultimate effective prestress is typically uniformly distributed throughout the component. Prior to the tensioning process of the RBPCP, the pile shaft concrete has cured. The lateral frictional resistance of the pile fluctuates along the depth of confining soil strata. During the tensioning process, part of the prestress is transferred to the foundation due to the lateral frictional resistance of the pile shaft. Consequently, the distribution mode of the effective prestress of the pile will differ from that of conventional prestressed concrete components tensioned on the ground.

To explore this mechanical phenomenon, a static uplift loading test on RBPCPs was carried out in a multistorey basement project in Hangzhou, and the corresponding prestress loss characteristics were analyzed.

### 4.2. Specimen design

Table 1 summarizes the stratigraphic parameters, and Fig 1 presents the configuration of test pile SZ1. The pile has a diameter (*D*) of 600 mm and an effective length (*L*) of 41.35m. The concrete strength grade for the pile is C50 (underwater), while the characteristic tensile strength of the prestressed steel strands is 2230MPa.

**Table 1:** Parameters of the soil layers.

Soil layer	*h* (m)	*γ*(kN/m^3^)	*E*_s_(MPa)	*c*(kPa)	*φ*	*ν*	*q*_sa_(kPa)
1: miscellaneous fill	2.95	18.0	8.00	8.0	15.0°	0.32	28
2−2: mucky clay	1.00	16.9	2.09	11.8	9.7°	0.37	28
3−1: silty clay	4.60	19.1	6.65	37.5	17.6°	0.31	60
3−2: silty clay	4.50	18.6	5.71	30.0	16.7°	0.31	55
4: clay	2.80	18.0	4.50	22.3	14.1°	0.34	40
5−1: silty clay	12.50	19.4	7.32	40.3	18.8°	0.32	66
6−1: silty clay	11.00	19.3	7.25	39.1	18.5°	0.31	84
6-2:medium sand	10.65	18.0	23.00	21.9	39.0°	0.30	136.8

In the test pile, segmental reinforcement was implemented. The upper two-thirds of the pile utilized 12C16 ordinary reinforcing bars and 12As15.2 RBPRs, whereas the lower one-third was reinforced with 8C16 ordinary reinforcing bars and 8As15.2 RBPRs. The test pile was reinforced using spiral stirrups of C6@250, which were modified to C6@100 within 4m from the top of the pile.

**Fig 5:**
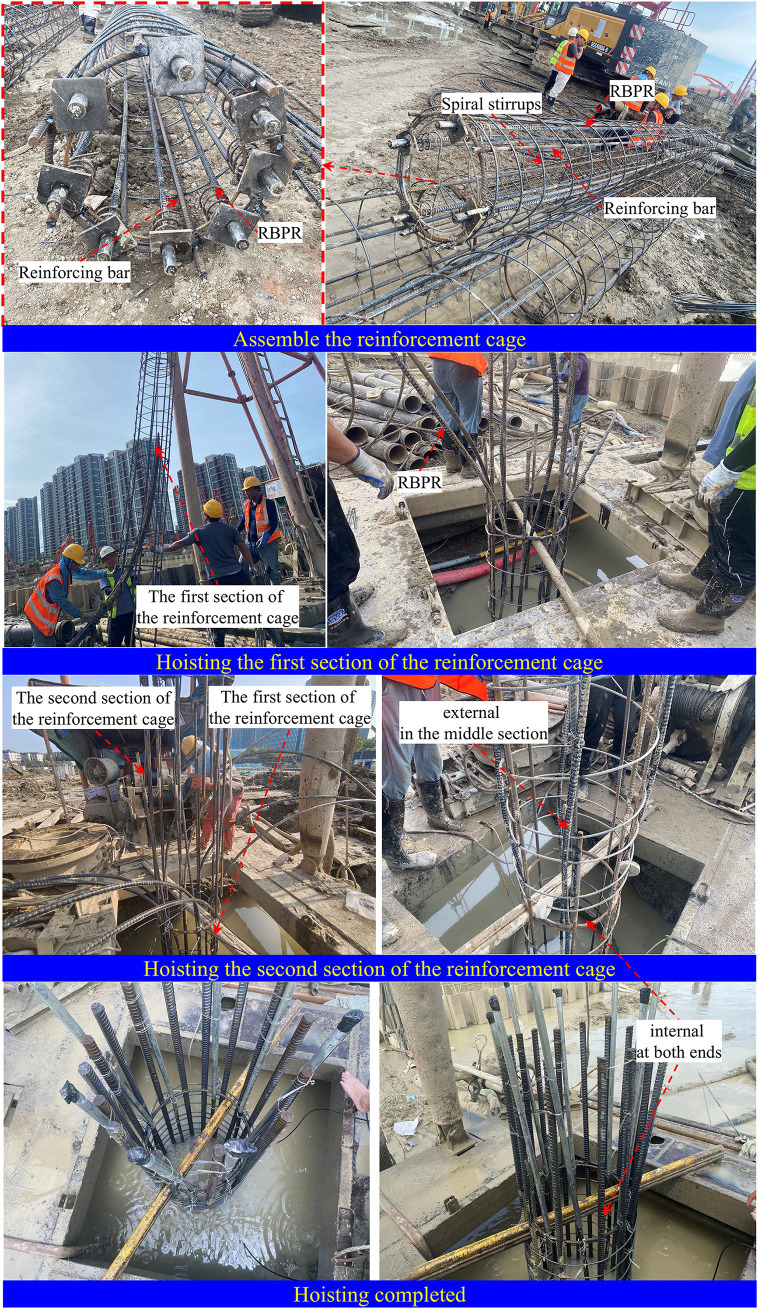
Procedure for installing the reinforcing cage for the RBPCP. (Original photographs taken by the authors).

Fig 5 illustrates the installation process of the reinforcing cage.

### 4.3. Experimental program

**Fig 6:**
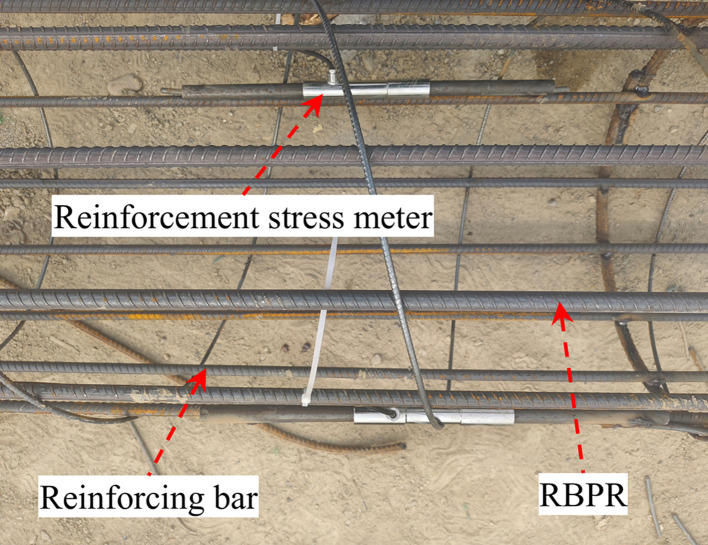
The installation of reinforcing bar stress meters (Original photographs taken by the authors).

**Fig 7:**
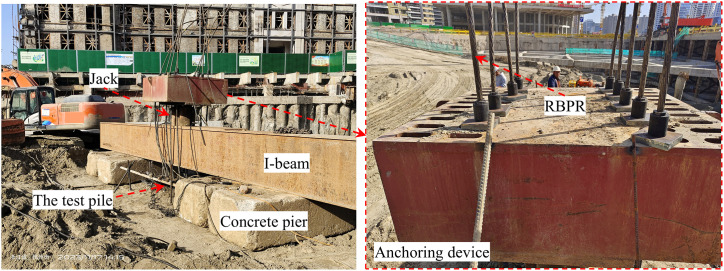
The testing of SZ1 (Original photographs taken by the authors).

Measuring points of the test pile were positioned at cross-sections corresponding to elevations of -1m, -6m, -11m, -16m, -21m, -26m, -31m, -36m and -41m in relation to the pile top. At each cross-section, two reinforcing bar stress meters were installed, as illustrated in Fig 6. The reinforcing bar stress meters have a strain range of ±1500μ*ε*, a measurement accuracy of ±0.5% full scale(F.S.), and a resolution of 0.1μ*ε*. Following the curing of the pile shaft concrete, a tensile force was exerted on the RPBPs, initially set at a modulus of 200kN and ultimately reaching a load of 2400kN, as shown in Fig 7.

### 4.4. Results and analysis

**Fig 8:**
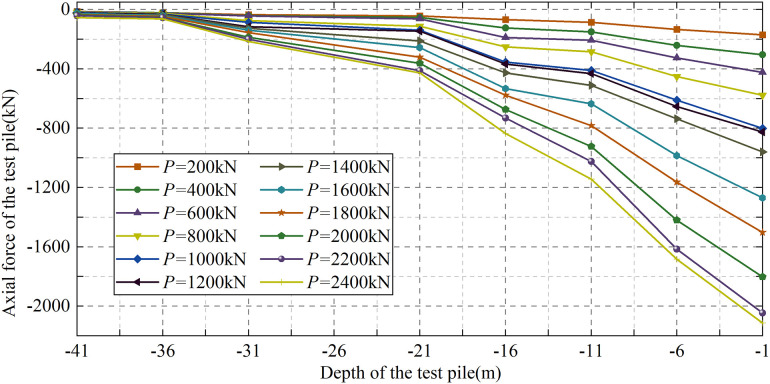
The axial force of the pile.

Fig 8 indicates the variation curves of the monitored axial force at each cross-section of SZ1 during the prestress tensioning and locking stages. The maximum tensile force (*P*) exerted on the prestressed steel strands is 2400 kN, and no reliable data was recorded at the depth of −26 m. Affected by pile shaft friction, the prestress established in the pile decreases gradually with increasing depth.

To intuitively describe the prestress loss due to soil constraints, the prestress loss ratio (*δ*_s_) is introduced:


$$\begin{array}{c} \delta_{s}=\frac{N_{\text{0}}-N_{l}}{N_{\text{0}}}\times 100\% \end{array}$$ (8)

Where *N*_0_ is the axial force of the pile at the depth of -1m (N); *l* denotes the depth of the pile (m); *N*_*l*_ indicates the axial force of the pile at the depth of *l* (N).

The prestress loss ratio (*δ*_s_) of each measuring point is detailed in Table 2. The upper section of the pile corresponds to a depth range from *l* = 0m to *l* = -14m, the middle section spans from *l* = -14m to *l* = -28m, and the lower section extends from *l* = -28m to *l* = −41.35m.

**Table 2:** The prestress loss ratio constrained by soil at each monitoring section.

Measuring points		*P =* 400kN	*P =* 800kN	*P* *=* 1200kN	*P* *=* 1600kN	*P* *=* 2000kN	*P* *=* 2400kN
Upper part	*l* = -6m	20.66%	21.66%	20.94%	22.46%	21.14%	20.39%
	*l* = -11m	50.49%	50.61%	47.70%	49.88%	48.72%	45.93%
Middle part	*l* = -16m	59.34%	56.33%	55.57%	58.08%	62.60%	60.50%
	*l* = -21m	82.62%	80.59%	82.32%	79.75%	79.91%	79.85%
Lower part	*l* = -31m	86.56%	87.18%	86.08%	89.13%	89.68%	89.83%
	*l* = -36m	92.79%	95.49%	96.13%	96.77%	97.11%	97.07%
	*l* = -41m	95.08%	96.88%	97.22%	98.03%	98.00%	97.40%

The soil-constrained prestress loss ratio (*δ*_s_) of the pile is observed to be 21% at a depth of 6m in the upper section, increasing to 48% at 11m. At a depth of 16m in the middle section, the rate is 58%, rising to 80% at 21m. Below 30m, the soil-constrained prestress loss ratio (*δ*_s_) at the pile bottom exceeds 90%.

An excessively high prestress loss ratio (*δ*_s_) may fail prestress establishment, consequently impacting the safety of the project. The superior mechanical properties of the upper soil layer in this test resulted in a pronounced pile-soil interaction effect, leading to a significant prestress loss in the pile. In conclusion, it is essential to perform an investigation into the safety performance of the RBPCP.

## 5. Research on the safety performance of RBPCP

The test results presented in Section 4 indicate that the effective prestress of the test pile SZ1 diminishes as the depth increases. Given that the initially tensile force is *N*, the effective prestressing force *N*_p_(*x*) at each cross-section of the pile can be determined using the following equations:


$$\begin{array}{c} N\geq N_{\text{qs}},N_{\text{p}}\left(x\right)=N-u\sum_{i=1}^{m-1}\int_{\text{0}}^{l_{i}}q_{\text{s}i\left(x\right)\text{a}}dx-u\int_{\text{0}}^{l_{m}}q_{\text{s}m\left(x\right)\text{a}}dx \end{array}$$ (9)

$$\begin{array}{c} N<N_{\text{qs}},N_{\text{p}}\left(x\right)=N\left(1-\frac{u\sum_{i=1}^{m-1}\int_{\text{0}}^{l_{i}}q_{\text{s}i\left(x\right)\text{a}}dx-u\int_{\text{0}}^{l_{m}}q_{\text{sm}\left(x\right)\text{a}}dx}{N_{\text{qs}}}\right) \end{array}$$ (10)

$$\begin{array}{c} N_{\text{qs}}=u\sum_{i=1}^{n}\int_{\text{0}}^{l_{i}}q_{\text{s}i\left(x\right)\text{a}}dx \end{array}$$ (11)

Where *N*_qs_ represents the pile shaft friction (N); *u* denotes the pile circumference (m); *i* indicates the soil layer number; *m* refers to the soil layer number of the calculated cross-section; *l*_*i*_ signifies the thickness of the *i*-th soil layer (m); *l*_m_ is the thickness of the calculated cross-section of its soil layer (m); *n* is the total number of soil layers; and *q*_si(*x*)a_ is the characteristic value of lateral friction resistance of piles at the depth of *x* (kPa).

As the pile shaft resistance increases with depth, it is logical to conclude that the required prestress at the end section of the pile is lower than that at the top section. In this section, analysis of required prestress and reserve prestress for test pile SZ1 was presented, aiming to evaluate the safety performance of the RBPCP.

### 5.1. Model establishment and validation

**Fig 9:**
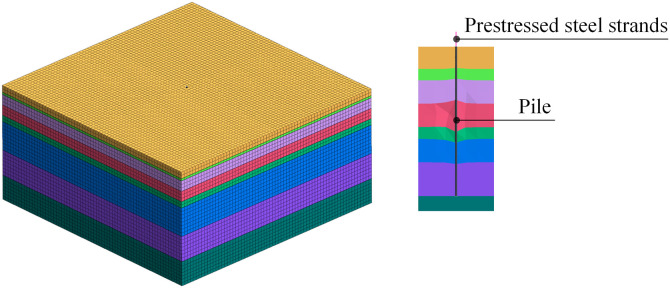
The finite element model of the pile SZ1.

A three-dimensional finite element model of the test pile SZ1 was created using Midas/GTX NS, as shown in Fig 9. The soil layers were established using solid elements. In contrast, the pile body and prestressed steel strands were modeled with beam elements. The interface between the pile and soil was simulated using interface pile elements. The model was simplified by omitting the spiral stirrups and standard reinforcing bars in the pile. The overall dimensions of the model were established at 100m × 100m × 50m to reduce the impact of boundary effects. All lateral boundaries of the soil were constrained in the normal direction, and the model base was fully fixed. The lower ends of the steel strands were anchored to the pile bottom to replicate the tensioning end. Since the primary focus of this section was the effective prestress in the pile, the subsequent bonding between the steel strands and the pile concrete was not temporarily simulated.

The modified Mohr-Coulomb model was adopted in the soil, with the parameters detailed in Table 1. The material model for both the pile and the steel strands is characterized as a linear elastic material. The pile has a diameter of 600 mm and a length of 41.35 m. The steel strands have a diameter of 15.2 mm and a length of 41.85 m. The process of calculation was conducted as described below:

(1) Activate the soil elements and boundary conditions, apply gravity, and perform the in-situ stress balance calculation;
(2) Activate the cast-in-place pile and prestressed steel strands, then define a rigid connection between the lower ends of the strands and the pile base;
(3) Apply a vertically upward tension force (*P*) at the free ends of the steel strands to simulate the prestress tensioning procedure; concurrently, apply a vertically downward reaction force (*N*) at the top of the pile to emulate the prestress locking mechanism. *N* has a magnitude that is equal to *P* but acts in the opposite direction, as detailed in Table 3.

**Table 3:** Loading protocol.

Condition	*P*(kN)	*N*(kN)	Condition	*P*(kN)	*N*(kN)
1	200	−200	7	1400	−1400
2	400	−400	8	1600	−1600
3	600	−600	9	1800	−1800
4	800	−800	10	2000	−2000
5	1000	−1000	11	2200	−2200
6	1200	−1200	12	2400	−2400

**Fig 10:**
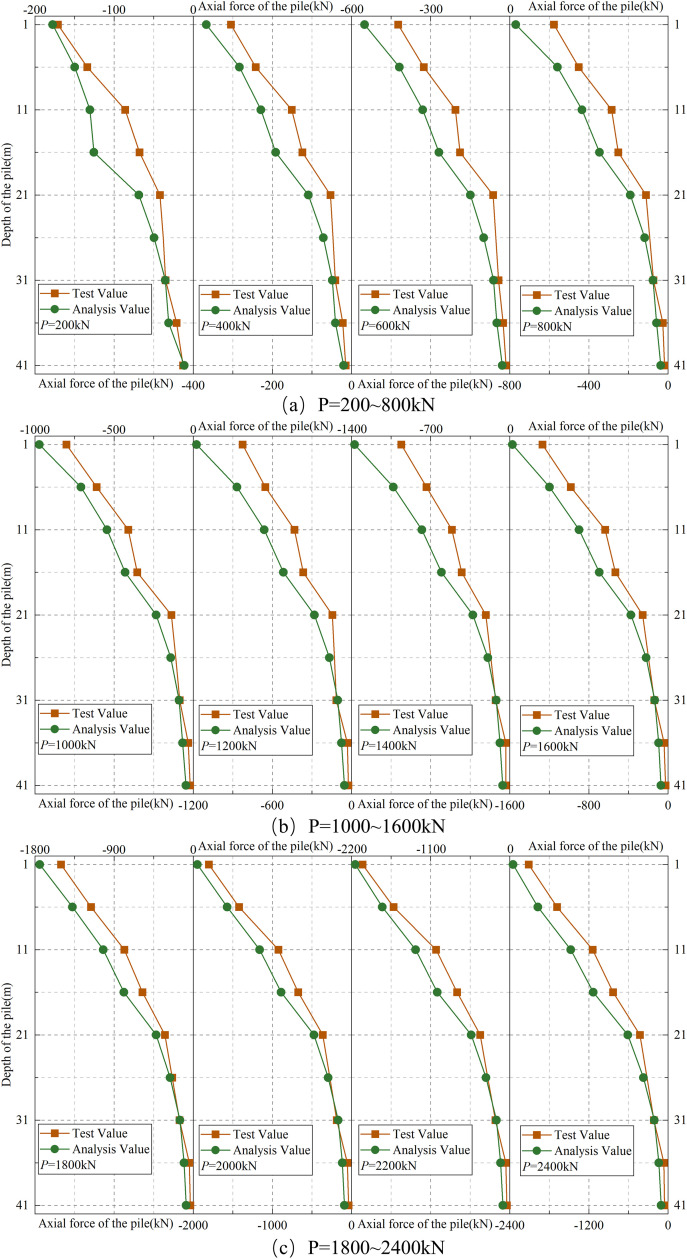
The pile axial force comparison of the calculation and test results.

The comparison between the calculation and test results of the axial force of the pile under each condition is shown in Fig 10. The effective prestress of the pile is observed to decrease as the depth increases. The axial force for each cross-section derived from numerical analysis is marginally greater than the results obtained from testing. The distinction primarily pertains to the prestress loss resulting from the deformation and friction of the anchor device during the testing process. Since the anchor device received simplification through the application of a reaction force in the numerical model, resulting in the omission of this portion of the prestress loss from the calculations.

Taking conditions 8 and 12 as examples, Table 4 lists the soil-restrained prestress loss ratios at different depths along the pile shaft. The soil-constrained prestress loss ratio varies from 0% to 47% along the upper pile shaft, rises to 47% ~ 86% in the middle section, and reaches approximately 86% ~ 95% within the lower section. The calculated results agree well with experimental data. In terms of variation regularity, the calculation results agree well with test results regarding both magnitude and distribution characteristics, verifying the validity of the finite element model for test pile SZ1 for safety performance evaluation.

**Table 4:** The soil-constrained prestress loss ratios under condition 8 and 12, *δ*_s_(%).

Condition	*P*	Upper part of the pile			Middle part of the pile			Lower part of the pile		
		*l* = -1m	*l* = -6m	*l* = -11m	*l* = -16m	*l* = -21m	*l* = -26m	*l* = -31m	*l* = -36m	*l* = -41m
8	1600 kN	2.31	20.27	41.20	56.10	74.11	85.24	91.46	93.98	95.48
12	2400 kN	1.44	13.39	35.57	52.38	71.90	84.41	91.29	94.19	95.58

### 5.2. Analysis of required prestress and reserve prestress

**Fig 11:**
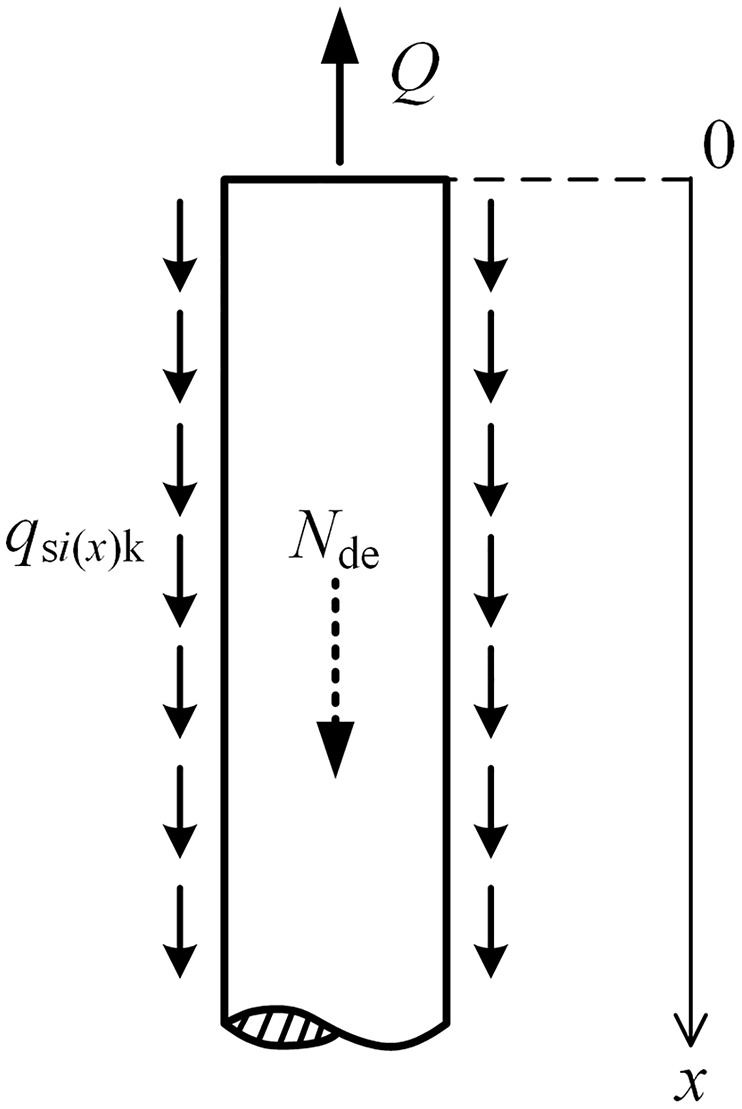
Schematic diagram of the demand pressure analysis.

The uplift load *Q* is applied at the pile top to simulate water buoyancy. Pile self-weight is neglected, and the force analysis of the pile is shown in Fig 11.

The required prestressing force to ensure the pile remains in compression can be determined from the following equation:


$$\begin{array}{c} N_{de}\left(x\right)=Q-u\sum_{i=1}^{m-1}\int_{\text{0}}^{l_{i}}q_{\text{s}i\left(x\right)\text{a}}dx-u\int_{\text{0}}^{l_{m}}q_{\text{sm}\left(x\right)\text{a}}dx \end{array}$$ (12)

In the initial model established in Section 5.1, the uplift load *Q* was gradually applied to 2400kN in steps of 100kN, allowing for the calculation of the required prestressing force of the pile under varying uplift loads.

**Fig 12:**
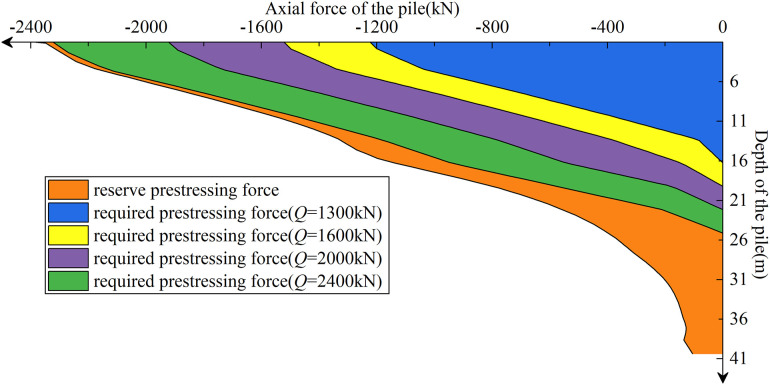
Comparison graphs illustrating the required prestressing force and reserve prestressing force across various uplift load scenarios.

The pile axial force subjected to the tension load *P* = 2400 kN in Section 5.1 were used as the reserve prestressing force. Fig 12 illustrates the comparison curves of the required prestressing force and reserve prestressing force of the pile under varying uplift loads *Q*. The following conclusions can be drawn:

(1) There is a substantial requirement for prestressing force within the upper and middle pile shaft, and the region needing prestressing force expands progressively with increasing uplift load.
(2) For *Q* values less than 2400kN, the reserve prestressing force in the pile adequately covers the required prestressing force, providing a degree of redundancy to maintain the pile in full compression;
(3) At *Q* equal to 2400kN, the reserve prestressing force in the pile matches the required prestressing force, placing the upper cross-section of the pile in a tension-compression balance state.

In summary, when the tensioning load is no less than the designed uplift bearing capacity, the prestressing force developed in the upper shaft of the RBPCP is sufficient to maintain full‑section compression of the pile. Only a marginal prestressing force is required for the middle and lower pile segments, and full‑section compression can still be guaranteed even if the prestress loss ratio exceeds 90%. Consequently, the RBPCP exhibits satisfactory structural safety performance.

## 6. Summary and conclusions

This paper presents an overview of the retard-bonded prestress cast-in-place pile (RBPCP) and the associated load test equipment. Based on experimental research, the analysis of prestress loss in the RBPCP was conducted. Further, the required prestress and reserve prestress were quantified to assess the structural safety performance of RBPCP. The key findings are summarized below:

(1) In comparison to traditional cast-in-place piles, RBPCPs exhibit superior crack resistance, leading to reduced construction costs for individual piles and broader applicability in engineering projects;
(2) It is recommended to implement the arrangement of “external in the middle section and internal at both ends” for RBPR to enhance construction efficiency while maintaining the bearing capacity of RBPCPs.
(3) The two-stage loading method, along with the novel test equipment developed in this paper, should be utilized for conducting static uplift load tests on RBPCPs.
(4) The soil surrounding the pile will cause significant prestress loss which increases continuously from the top to the bottom of the pile. The prestress loss ratio at the top of the pile is close to 50%, while the prestress loss ratio in the middle and lower sections is generally over 90%.
(5) The effective prestress of the cross-section of RBPCPs diminishes progressively with increasing depth due to pile-soil interaction. When the tension load meets or exceeds the design value of the uplift bearing capacity, it can be ensured that the entire cross-section of the pile remains in compression. The required prestress near the base of the pile is minimal, and any prestress loss does not impact the bearing capacity of the lower section of the pile.

## Supporting information

S1 Data S1 Data. (XLSX)
